# Dihydroartemisinin and its derivative induce apoptosis in acute myeloid leukemia through Noxa-mediated pathway requiring iron and endoperoxide moiety

**DOI:** 10.18632/oncotarget.3336

**Published:** 2015-01-21

**Authors:** Xuan Zhao, Hang Zhong, Rui Wang, Dan Liu, Samuel Waxman, Linxiang Zhao, Yongkui Jing

**Affiliations:** ^1^ Department of Pharmacology, Shenyang Pharmaceutical University, Shenyang, China; ^2^ Department of Chemical Synthesis, Shenyang Pharmaceutical University, Shenyang, China; ^3^ Department of Medicine, The Tisch Cancer Institute, Icahn School of Medicine at Mount Sinai, New York, NY, USA

**Keywords:** Dihydroartemisinin, Noxa, Apoptosis, ABT-737, Acute myeloid leukemia

## Abstract

Anti-apoptotic protein Mcl-1 plays an important role in protecting cell from death in acute myeloid leukemia (AML). The apoptosis blocking activity of Mcl-1 is inhibited by BH3-only protein Noxa. We found that dihydroartemisinin (DHA) and its derivative X-11 are potent apoptosis inducers in AML cells and act through a Noxa-mediate pathway; X-11 is four-fold more active than DHA. DHA and X-11-induced apoptosis is associated with induction of Noxa; apoptosis is blocked by silencing Noxa. DHA and X-11 induce Noxa expression by upregulating the transcription factor FOXO3a in a reactive oxygen species-mediated pathway. Interfering with the integrity of the endoperoxide moiety of DHA and X-11, as well as chelating intracellular iron with deferoxamine, diminish apoptosis and Noxa induction. AML cells expressing Bcl-xL, or with overexpression of Bcl-2, have decreased sensitivity to DHA and X-11-induced apoptosis which could be overcome by addition of Bcl-2/Bcl-xL inhibitor ABT-737. DHA and X-11 represent a new group of AML cells-apoptosis inducing compounds which work through Noxa up-regulation utilizing the specific endoperoxide moiety and intracellular iron.

## INTRODUCTION

Acute myeloid leukemia is a disease of malignant proliferation of hematopoietic cells with disrupted differentiation and apoptotic program. Chemotherapy remains the main therapy and the therapeutic outcome of AML has not been significantly improved during the last forty years. Molecular and cellular genetic analyses in AML revealed many potential signaling pathways which have been used to develop therapeutic agents [1, 2], but so far only limited clinic activities have been achieved [3, 4]. It seems that agents selectively inducing cell death would be more effective for AML treatment. Mitochondrial-mediated apoptosis, controlled by the anti-apoptotic protein Bcl-2 family, Bcl-2, Bcl-xL and Mcl-1 [5], was thought to be the main mechanism of AML cell killing by chemotherapy. Increased levels of Bcl-2, Bcl-xL and/or Mcl-1 have been found to predict poor prognosis of AML patients to chemotherapy [6-8]. Although agents targeting Bcl-2/Bcl-xL have been developed [9], the effect of Bcl-2/Bcl-xL inhibitors was hindered by basal and/or increased expression of Mcl-1 [10]. Molecular studies revealed that Mcl-1 plays an even more important role than Bcl-2/Bcl-xL in protecting AML cells from apoptosis [11] and, therefore, agents inhibiting Mcl-1 need to be developed.

**Figure 1:**
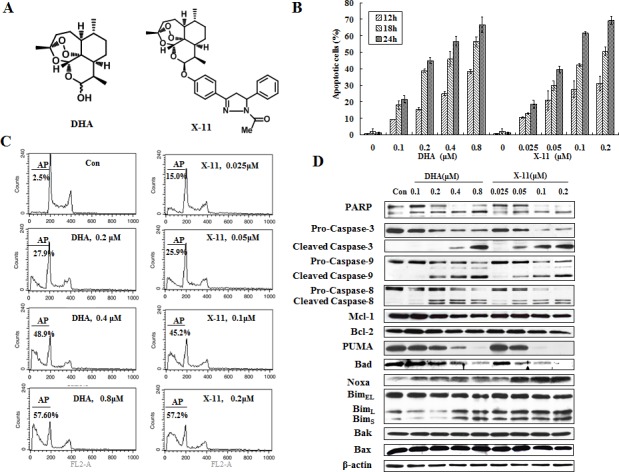
X-11 is more potent than DHA in apoptosis induction in HL-60 cells (A), chemical structures of DHA and X-11. (B), dose- and time-dependent apoptosis induction in HL-60 cells. Cells were treated with DHA and X-11 at the indicated concentrations for 12, 18 and 24 h. Percentages of apoptotic cells were determined based on morphological changes using a fluorescence microscope after staining with AO and EB. (C), percent of apoptotic HL-60 cells treated with DHA and X-11 for 24 h at the indicated concentrations. Apoptosis measured using staining with PI followed by FACS analysis and identification of the SubG1 population. AP, apoptotic cells; Con, control. (D), Western blot analyses of apoptosis-related proteins in HL-60 cells treated with DHA and X-11 for 24 h at the indicated concentrations. The relative levels of the proteins were determined by probing with specific antibodies. β-actin served as loading control.

Artemisinin is a sesquiterpene lactone isolated from the sweet wormwood *Artemisia annua L* and is being used as an antimalarial agent [12]. Artemisinin, its ester and ether have been reported to have antitumor effects [13]. The anti-proliferative effects of these artemisinin analogues were tested in National Cancer Institute (NCI) 60 cell line panel which were clustered into three response groups with leukemia cells being the most responsive [13, 14]. Dihydroartemisinin (DHA) is an active metabolite of arteminisin analogues and has been shown to induce apoptosis in AML cells [15, 16]. To improve the anti-leukemia activity of DHA we have synthesized a series of derivatives substituted with a chalcone or a piperazine [17]. DHA derivatives substituted with a chalcone showed improved anti-proliferative ability over DHA and also induced apoptosis in AML HL-60 cells [17]. We also found that DHA derivatives substituted with a piperazine were more potent than DHA in induction of apoptosis in HL-60 cells. Although several factors have been found to contribute to DHA-induced apoptosis, the mechanism of action is unclear. In this study we selected one of the most active derivatives, X-11 (10-O-[4-(1-acetyl-5-phenyl-4, 5-dihydropyrazol-3-yl) phenyl]-(10S)-dihydroartemisinin, Fig. 1A), and DHA to compare their apoptosis induction abilities and to investigate the mechanism of action in several AML cell lines. We found that up-regulation of BH3-only protein Noxa, by inactivating Mcl-1, plays an important role in DHA and X-11-induced apoptosis. This effect relies on the endoperoxide moiety of DHA and X-11 as well as the intracellular iron of AML cells.

## RESULTS

### X-11 induces apoptosis in HL-60 cells more potently than DHA and this effect is associated with the induction of Noxa

HL-60 cells were treated with several concentrations of DHA or X-11 for 12, 18 and 24 h and apoptotic cells were measured based on morphological changes after staining with acridine orange (AO) and ethidium bromide (EB). X-11 was more potent than DHA in inducing apoptosis (Fig. 1B). The comparative levels of apoptotic cells after treatment with DHA or X-11 at different concentrations were confirmed by measuring fragmented DNA (hypodiploid DNA) using FACS (Fig. 1C). While about 57% of HL-60 cells underwent apoptosis after treatment with 0.2 μM X-11 for 24 h, a 4-fold higher concentration of DHA was required to induce the same amount of apoptotic cells (Fig. 1C).

To determine the mechanism of apoptosis induction by DHA and X-11 treatment, the levels of apoptosis-related proteins were investigated in HL-60 cells treated with these two compounds. Altered levels of cleaved PARP in cells treated with DHA and X-11 corresponded to levels of cleaved caspase-3, caspase-8 and caspase-9, suggesting that all three caspases participated in apoptosis induction (Fig. 1D). Although, there was a report showing that caspase-8 was activated in HL-60 cells after DHA treatment, the activation of caspase-9 was not determined [15]. In a separated report it was found that a sub-clone of Jurkat cells defective in caspase-8 expression was responsive to DHA-induced apoptosis [18]. We compared the apoptosis induction ability of DHA and X-11 in Jurkat sub-clones, I 9.2 cells with defective caspase-8 and A3 cells expressing caspase-8. Both cell lines were equally sensitive to DHA- and X-11-induced apoptosis (Supp Fig. 1A); in both lines apoptosis was associated with the activation of caspase-9 (Supp Fig. 1B), indicating that a mitochondrial-mediated apoptotic pathway plays a more important role than death receptor-mediated pathway. Of note is the fact that much higher concentrations of DHA and X-11 were needed to induce apoptosis in both I 9.2 and A3 cell lines as compared to that used in HL-60 cells (Supp Fig. 1, Fig. 1). The mitochondrial apoptotic pathway leading to caspase-9 activation is controlled by anti-apoptotic proteins Bcl-2, Bcl-xL and Mcl-1, pro-apoptotic proteins Bax and Bak, as well as the BH3-only proteins Bad, Bim, PUMA and Noxa [19, 20]. The levels of those proteins were measured in HL-60 cells treated with DHA and X-11. Previously we reported that HL-60 cells did not express Bcl-xL and that decreasing the Mcl-1 protein level could induce apoptosis in HL-60 cells [21, 22]. Neither Mcl-1 level, nor Bcl-2, Bax or Bak were changed after treatment with DHA and X-11 (Fig. 1D). HL-60 cells express high levels of Bad, Bim and PUMA, but lower levels of Noxa. The levels of Bad and PUMA were decreased after treatment with DHA and X-11. Noxa, Bim_L_ and Bim_S_, but not Bim_EL_, were induced by treatment with DHA and X-11 in HL-60 cells. Since Noxa, Bim_L_ and Bim_S_ were also induced in Jurkat A3 and I 9.2 sub-clones treated with higher concentrations of DHA and X-11 (Supp Fig. 1B), these data suggest that Noxa and/or Bim-mediated mitochondrial apoptotic pathway accounts for DHA and X-11-induced apoptosis.

### Superoxide (O_2_^−^), but not H_2_O_2_, plays an important role in DHA and X-11-induced apoptosis in HL-60 cells

Reactive oxygen species (ROS) have been reported to be involved in DHA-induced apoptosis in Jurkat and melanoma cells [18, 23], but not in HL-60 cells [15]. The levels of ROS in HL-60 cells treated with DHA and X-11 were measured using a H_2_O_2_-sensitive fluorescent probe DCFH-DA. DHA and X-11 treatment for 6, 9 and 15 h increased the amount of H_2_O_2_ (Supp Fig. 2A). Although pretreatment with either *N*-acetylcysteine (NAC) or catalase (CAT) decreased H_2_O_2_ accumulation caused by DHA and X-11 treatment (Supp Fig. 2B), neither NAC nor CAT blocked DHA and X-11-induced apoptosis (Supp Fig. 2C). Pretreatment with NAC and CAT altered only very minimally the activation of caspase-3 and -9 as well as the up-regulation of Noxa and Bim (Supp Fig. 2D). Therefore, these data are consistent with the previous report showing that DHA-induced apoptosis could not be blocked by NAC in HL-60 cells [15].

**Figure 2:**
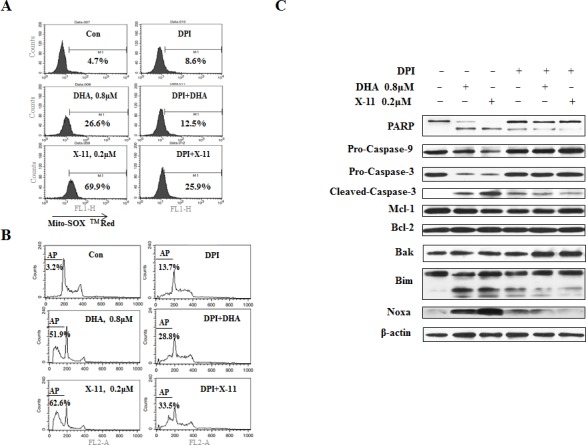
Superoxide (O) is induced by DHA and X-11 and contributes to apoptosis HL-60 cells pretreated with 1 μM DPI or not pre-treated, were exposed to 0.8 μM DHA or 0.2 μM X-11 for 15 h. The intracellular O_2_^−^ content was determined by adding 5 μM MitoSOX^TM^ Red followed by FACS analysis. The peak shift to the right indicates increase in the levels of O_2_^−^ content (A). The effects of DPI on DHA and X-11-induced apoptosis were determined by PI staining followed by FACS analysis (B). Cleavage of PARP, caspase-3, -9, as well as Noxa and Bim levels were determined by Western blot using specific antibodies (C).

To find out if O_2_^−^ has a role in DHA/X-11-induced apoptosis, O_2_^−^-sensitive fluorogenic dye MitoSOX^TM^ Red was used to measure O_2_^−^ levels. Treatment with 0.2 μM X-11 and 0.8 μM DHA increased the levels of O_2_^−^ (Fig. 2A). Diphenyleneiodonium chloride (DPI), an O_2_^−^ inhibitor [24], attenuated the DHA and X-11 treatment-increased O_2_^−^ levels of HL-60 (Fig. 2A). Correlated with the inhibition of O_2_^−^ accumulation, DHA and X-11-induced DNA fragmentation was attenuated by DPI (Fig. 2B). DPI also inhibited the cleavage of PARP, caspase-3 and -9, as well as the up-regulation of Noxa and Bim_L_induced by DHA and X-11 treatment (Fig. 2C). These data suggest that O_2_^−^, but not H_2_O_2_, plays an important role in apoptosis induction and Noxa up-regulation of HL-60 cells treated with DHA and X-11.

### Iron is required for DHA/X-11-induced apoptosis in HL-60 cells

**Figure 3:**
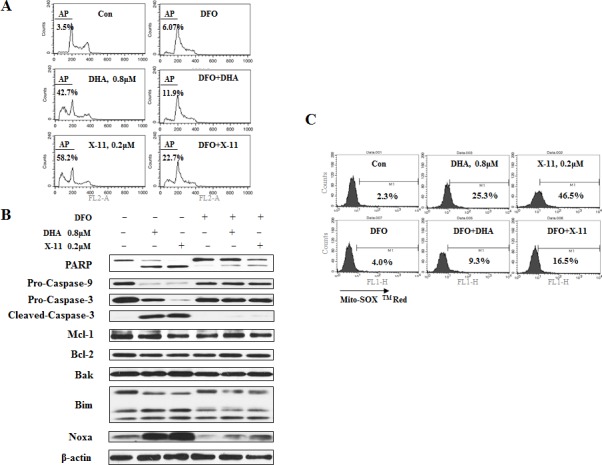
Iron is required for DHA and X-11-induce apoptosis and O_2_ production HL-60 cells were pretreated for 4 h with 100 μM DFO, the iron chelator, or not pretreated, and then treated with DHA/X-11 for 24 h at the indicated concentrations. Cells were stained with PI and apoptotic cells determined by FACS analysis (A). Apoptosis related protein levels were measured by Western blot analyses (B). The levels of O_2_^−^ were measured using MitoSOX^TM^ Red by FACS (C).

Several reports show that iron plays an important role in DHA-induced cell death [15, 25, 26]. To test the role of iron in the apoptosis induction by DHA and X-11, iron chelator, deferoxamine mesylate (DFO) was used. Pretreatment with DFO significantly suppressed DHA/X-11-induced apoptosis (Fig. 3A). Addition of 100 μM DFO decreased 0.8 μM DHA-induced apoptosis from 42.7% to 11.9% and 0.2 μM X-11-induced apoptosis from 58.2 to 22.7%. DFO blocked DHA and X-11-induced cleavage of PARP, caspase-3 and caspase-9, as well as the up-regulation of Noxa and Bim (Fig. 3B). Although addition of 100 μM Fe^2+^ or Fe^3+^ reversed the blockage of DFO on DHA/X-11-induced apoptosis, exogenous Fe^2+^/Fe^3+^ only minimally enhanced DHA/X-11-induced apoptosis (Data not shown). These data suggest that the intracellular iron content of HL-60 cells is sufficient to activate DHA and X-11. Since DFO also attenuated DHA and X-11-induced O_2_^−^ production (Fig. 3C), it seems that iron plays an essential role in apoptosis induction by DHA and X-11 in leukemia cells.

### At low concentrations of DHA and of X-11 endoperoxide bridge is required for apoptosis induction in leukemia cells

**Figure 4:**
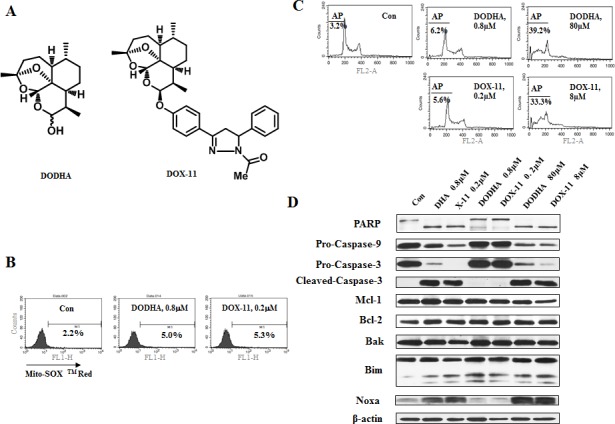
At low concentrations of DHA/X-11, an endoperoxide bridge is required for apoptosis induction (A), the chemical structures of DODHA and DOX-11 with the endoperoxide moiety containing only one oxygen. (B), the levels of O_2_^−^ in HL-60 cells treated with DODHA/DOX-11. HL-60 cells untreated or treated with 0.8 μM DODHA or 0.2 μM DOX-11 for 15 h were used to determine the intracellular O_2_^−^ content using MitoSOX^TM^ Red by FACS. (C & D), DODHA/DOX-11-induced apoptosis and protein changes. HL-60 cells were treated with DODHA, DOX-11, DHA or X-11 at the indicated concentrations for 24 h. The apoptotic cells were stained with PI and analyzed by FACS (C) and the levels of apoptosis related proteins were determined by Western blot analysis using specific antibodies (D).

The endoperoxide bridge of DHA interacts with iron to form carbon-centered radicals which cause cytotoxicity [16]. To test the requirement of the endoperoxide bridge in the apoptosis induction in cells treated with DHA and X-11, deoxygenated counterparts of DHA and X-11 (DODHA and DOX-11) were synthesized (Supp Fig. 3). In both compounds the endoperoxide bridge moiety contained only one oxygen (Fig. 4A). Unlike DHA and X-11, DODHA at 0.8 μM and DOX-11 at 0.2 μM neither increased the O_2_^−^levels (Fig. 4B) nor induced apoptosis (Fig. 4C), at 80 μM concentration of DODHA and 8 μM of DOX-11 both compounds increased the levels of Noxa (Fig. 4D). DODHA and DOX-11 have similar abilities as DHA and X-11 to induce Bim_L_ and Bim_S_ regardless of the concentrations used. These data suggest that the endoperoxide bridge of DHA and X-11 is the functional moiety for the induction of apoptosis and Noxa protein at lower concentrations.

### Noxa is a key mediator of DHA- and X-11-induced apoptosis in leukemia cells which is induced through a FOXO3a-mediated pathway

**Figure 5:**
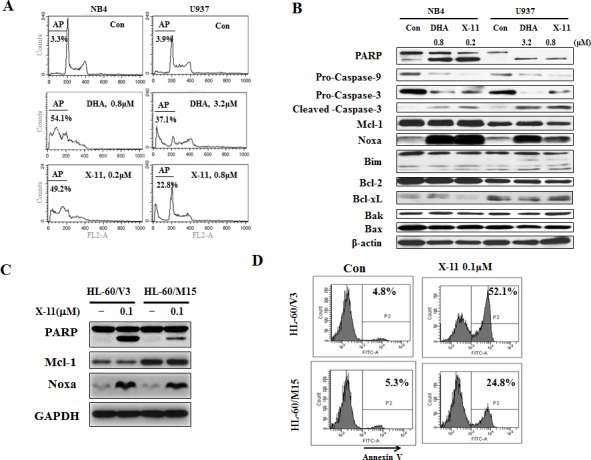
Induction of apoptosis by DHA/X-11 in AML cells with different levels of Bcl-xL and Mcl-1 NB4 and U937 cells were treated with DHA/X-11 at the indicated concentrations for 24 h. The apoptotic cells were determined by PI/FACS (A) and the protein levels were determined using Western blot analysis (B). HL60/M15 and HL60/V3 cells were treated with X-11 at 0.1 μM for 24 h and their extracts were used to measure the protein levels of Noxa and Mcl-1 with Western blotting (C) and the intact cells for percent of apoptosis by FACS after staining with annexin V (D).

The up-regulation of Noxa and Bim is associated with apoptosis induction in HL-60 cells in response to DHA and X-11 (Fig. 1D). Additional AML cell lines (NB4 and U937) were used to test apoptosis induction and Noxa/Bim up-regulation by DHA/X-11 treatment. While NB4 cells were as sensitive as HL-60 cells to DHA/X-11 treatment (Fig. 5A), U937 cells were less sensitive. Four-fold higher concentrations of either DHA (3.2 μM) or X-11 (0.8 μM) were required to induce apoptosis in U937 cells (Fig. 5A). The up-regulation of Noxa, but not Bim, was associated with the cleavage of PARP, caspase-3 and caspase-9 in both NB4 and U937 cell lines (Fig. 5B). Noxa is a pro-apoptotic protein that inactivates Mcl-1, but not Bcl-2 and Bcl-xL, and leads to apoptosis [27]. To test the role of Mcl-1 in Noxa induction-mediated apoptosis, HL-60/M15, HL-60 cells transfected with a Mcl-1 expression vector, and HL-60/V3, HL-60 cells transfected with an empty vector, were used to compare their responses to X-11-induced apoptosis. HL60/M15 cells contain higher levels of Mcl-1 than HL-60/V3 cells (Fig. 5C). Both sub-clones were treated with X-11 at 0.1 μM for 24 h. Although Noxa was induced to the same level by X-11 treatment in both sub-clones (Fig. 5C), apoptosis induction ability of X-11 was reduced in HL-60/M5 cells as determined by PARP cleavage (Fig. 5C) and staining with annexin V (Fig. 5D).

**Figure 6:**
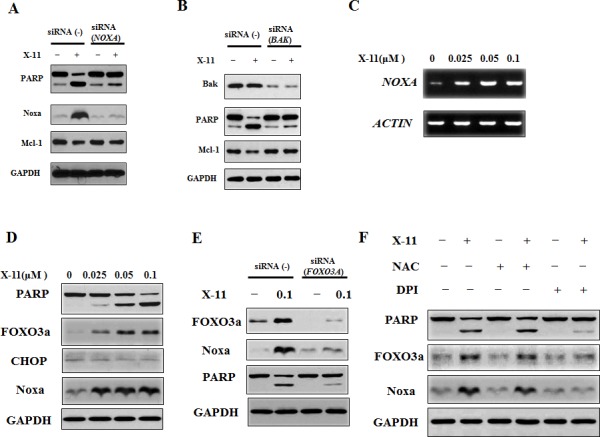
Noxa plays an important role in X-11-induced apoptosis and is induced through FOXO3a-mediated pathway A & B, NB4 cells were transfected with *NOXA* (A) *or BAK* (A) siRNA or a negative control siRNA, and after 18 h, treated with 0.2 μM X-11 for 18 h. The protein levels of PARP, Noxa, Mcl-1 and GAPDH were determined by Western blotting using specific antibodies. C & D, NB4 cells were treated with X-11 at the indicated concentrations for 24 h. The mRNA levels of *NOXA* were measured by RT-PCR (C) and the protein levels were measured by Western blot analysis (D). E, NB4 cells were transfected with *FOXO3A* siRNA or a negative control siRNA, and after 18 h, treated with 0.2 μM X-11 for 18 h. The protein levels of PARP, Noxa and GAPDH were determined. F, DPI, but not NAC, inhibited X-11-induced Noxa and FOXO3a. NB4 cells were pretreated with or without 1 μM DPI or 10 mM NAC followed by treatment with 0.2 μM X-11 for 15 h. The protein levels of PARP, Noxa, FOXO3a and GAPDH were determined.

NB4 and HL-60 cell lines are equally responsive to apoptosis induction and Noxa up-regulation by DHA/X-11 treatment. Noxa was silenced in NB4 cells. Silencing of Noxa blocked X-11-induced PARP cleavage without influencing the Mcl-1 level (Fig. 6A) and apoptosis (Supp Fig. 4). Noxa leads to Bak activation by competitive binding to Mcl-1 [28]. To test the role of Bak in the apoptosis induction by X-11 treatment, Bak was also silenced in NB4 cells. Similar to the silencing of Noxa, knocking down of Bak blocked X-11-induced PARP cleavage (Fig. 6B) and apoptosis (Supp Fig. 4). These data suggest that induction of Noxa plays an important role in the apoptosis induction. To test the signaling pathways through which Noxa is induced, the mRNA levels of *NOXA* were measured. X-11 treatment increased the mRNA levels in NB4 cells (Fig. 6C), suggesting the induction of Noxa is at the transcription level. It has been reported that Noxa can be induced by ROS through endoplasmic reticulum (ER) stress- and FOXO3a-mediated pathways [29-31]. We compared the levels of CHOP, a member of ER stress signaling, and FOXO3a protein levels and found that FOXO3a, but not CHOP, was induced after X-11 treatment (Fig. 6D). Silencing of FOXO3a blocked X-11-induced PARP cleavage and Noxa protein (Fig. 6E) as well as apoptosis (Supp Fig. 4). DPI, but not NAC, blocked X-11-induced apoptosis and Noxa in HL-60 cells (Fig. 2, Supp Fig. 2). Similarly, we found that DPI, but not NAC, blocked X-11-induced FOXO3a, Noxa and PARP cleavage in NB4 cells (Fig. 6F). These data suggest that FOXO3a is a mediator of O_2_^−^ and Noxa induction in AML cells treated with X-11.

### ABT-737 enhances DHA/X-11-induced apoptosis in U937 cells and in HL-60 cells with overexpression of Bcl-2

**Figure 7:**
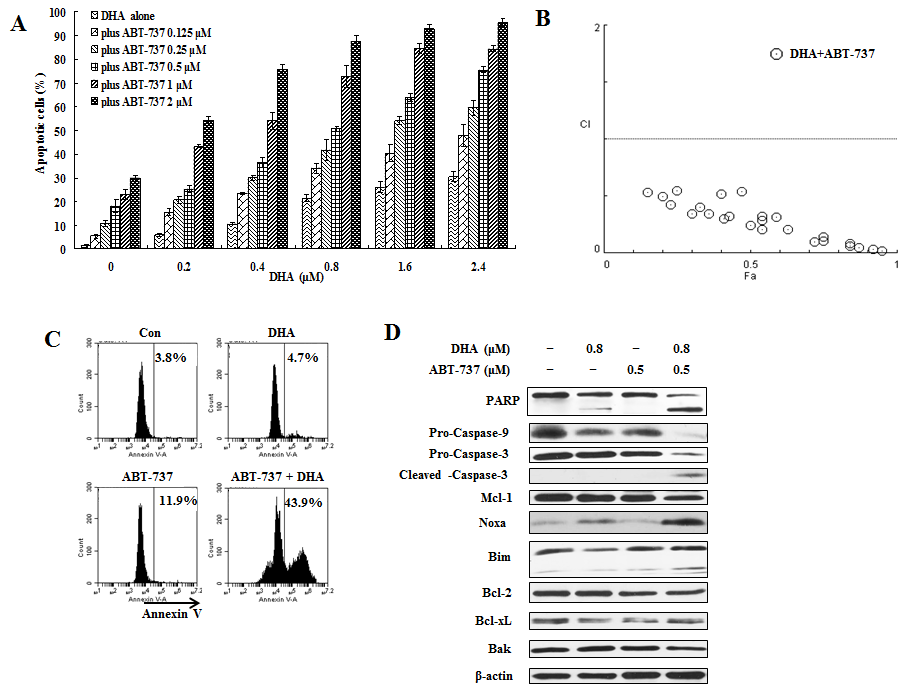
ABT-737 combined with DHA synergistically induce apoptosis in U937 cells U937 cells were treated with ABT-737 (0.125 to 2 μM), DHA (0.2 to 2.4 μM) or their combinations at different ratios for 24 h. Apoptotic cells were quantified based on morphologic changes by microscopic detection of AO/EB-stained cells (A). The nature of interaction between ABT-737 and DHA was characterized by median dose effect analysis using CompuSyn software. CI values of less than 1.0 (horizontal line) correspond to a synergistic interaction. Fa on the x- axis denotes the fraction affected (B). U937 cells were treated with 0.5 μM ABT-737, 0.8 μM DHA and their combination for 24 h. The apoptotic cells were determined by FACS after staining with annexin V-FITC (C) and the relative levels of indicated proteins were determined by Western blotting using specific antibodies (D).

**Figure 8:**
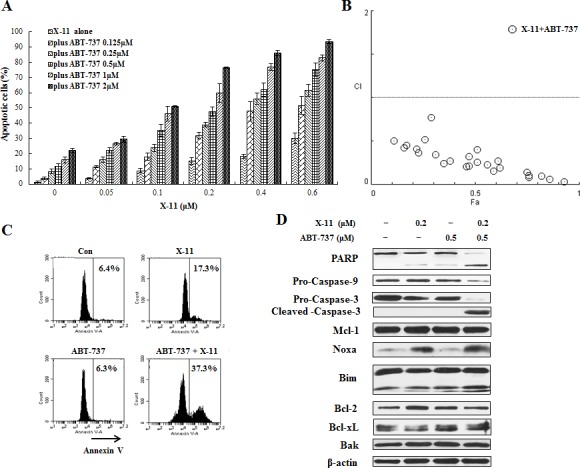
ABT-737 combined with X-11 synergistically induce apoptosis in U937 cells U937 cells were treated with ABT-737 (0.125 to 2 μM), X-11 (0.05 to 0.6 μM) or their combinations at different ratios for 24 h. Apoptotic cells were quantified based on morphologic changes by microscopic detection of AO/EB-stained cells (A). The nature of interaction between ABT-737 and X-11 was characterized by median dose effect analysis using CompuSyn software. CI values of less than 1.0 (horizontal line) correspond to a synergistic interaction (B). U937 cells were also treated with 0.5 μM ABT-737, 0.2 μM DHA and their combination for 24 h to determine the apoptosis induction by FACS after staining with annexin V-FITC (C) and protein regulation by Western blotting analysis (D).

U937 cells express high levels of Bcl-xL (Fig. 5B) [32]. Since Bcl-xL also inhibits Bak, an effect not antagonized by Noxa [33], the lesser sensitivity of U937 cells to DHA and X-11 treatment is probably due to their expression of Bcl-xL. ABT-737 is a small molecule inhibitor of Bcl-2/Bcl-xL proteins and induces apoptosis in AML cells [34]. The combined effect of DHA with ABT-737 was tested by treating U937 cells with different concentrations of DHA alone, and in combination with ABT-737 at different ratios, and apoptosis induction rates were determined by morphological observation after staining with AO/EB (Fig. 7A). Using Compusyn software, the combined apoptotic effects of DHA with ABT-737 were analyzed for synergy. CI values were calculated for different dose-effect levels based on parameters derived from median-effect plots of DHA alone, ABT-737 alone, or their combinations. As shown in Fig. 7B, simultaneous exposure of U937 cells to ABT-737 (0.125-2 μM) and DHA (0.2-2.4 μM) showed CIs of less than 1, indicating synergistic effects. The synergistic apoptotic effect was confirmed in U937 cells using FACS analysis of annexin V stained cells after treatment with 0.5 μM ABT-737 and 0.8 μM DHA. While individually, each of the compounds produced only <11% apoptotic cells, the combined treatment increased apoptosis to approximately 44% (Fig. 7C). DHA at 0.8 μM induced Noxa, but not PARP cleavage. Addition of ABT-737 enhanced cleavage of PARP and caspase-3 as well as Noxa level (Fig. 7D). Similar data were observed for U937 cells treated with ABT-737 (0.125-2 μM) in combination with X-11 (0.05-0.6 μM), in which synergistic apoptotic effects were obtained with CIs less than 1 were detected (Fig. 8A, 8B). The combined treatment of 0.5 μM ABT-737 with 0.2 μM X-11 induced approximately 38% apoptotic cells while individually, each compound produced only <17% (Fig. 8C). X-11 was more potent than DHA in Noxa induction which was not further enhanced by ABT-737 (Fig. 8D). These data suggest that the inhibition of Bcl-xL and/or Bcl-2 by ABT-737 together with Noxa induction by DHA/X-11 account for their synergistic apoptotic effect.

**Figure 9:**
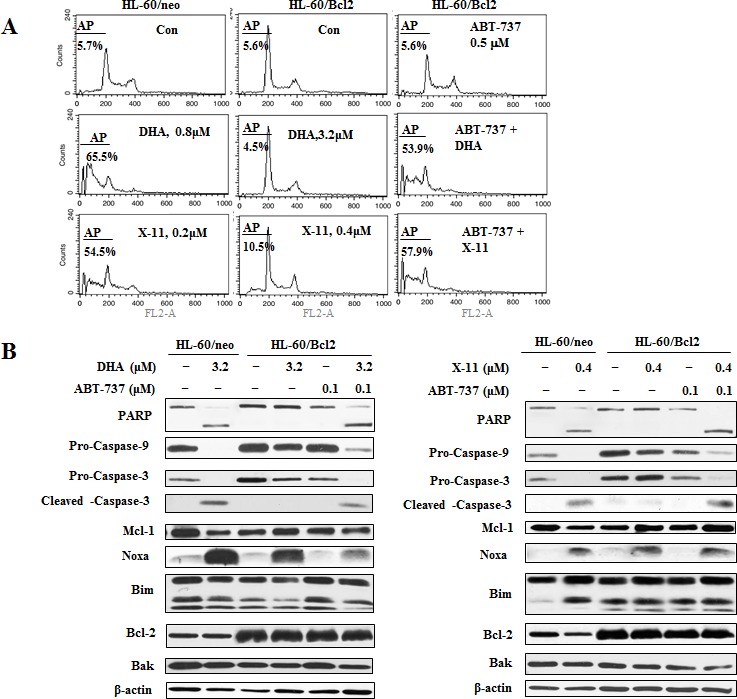
The combined effects of DHA/X-11 with ABT-737 in HL-60 cells which overexpress Bcl-2 HL-60/neo cells, (transfected with an empty vector), and HL-60/Bcl2, (transfected with a Bcl-2 expression vector), were treated with either DHA and X-11 alone or in combination with ABT-737 for 24 h. Percentages of apoptotic cells were determined using FACS after staining with PI (A) and the apoptosis related proteins were measured by Western blot analysis (B).

Increased levels of Bcl-2 were shown to be associated with resistance and relapse in anti-AML therapy [35, 36]. To test if the increased levels of Bcl-2 in HL-60 cells correspond to decreased response to DHA and X-11, clones of HL-60 transfected with either Bcl-2 (HL-60/Bcl2) or an empty vector (HL-60/neo) were tested. While HL-60/neo cells were as sensitive as the parental HL-60 cells HL-60/Bcl2 cells were less sensitive to DHA/X-11-induced apoptosis. While DHA at 0.8 μM and X-11 at 0.2 μM induced approximately 60% of HL-60/neo cells to undergo apoptosis, even at the higher concentrations, (DHA at 3.2 μM and X-11 at 0.4 μM), the compound induced apoptosis in only less than 10% of HL-60/Bcl2 cells (Fig. 9A), indicating that overexpression of Bcl-2 causes resistance to DHA/X-11. ABT-737 at a concentration of 0.1 μM alone did not induce apoptosis but enhanced apoptosis of HL-60/Bcl2 cells treated with 3.2 μM DHA or 0.4 μM X-11 (Fig. 9A). DHA at 3.2 μM and X-11 at 0.4 μM increased the levels of Noxa in both HL-60/neo and HL-60/Bcl2 cells, but cleavage of PARP and caspase-3 only occurred in HL-60/neo cells (Fig. 9B). Addition of ABT-737 together with DHA or X-11 induced cleavage of PARP and caspase-3 in HL-60/Bcl2 cells (Fig. 9B). These data suggest that increased levels of Bcl-2 decrease cell sensitivity to DHA and X-11 and this can be overcome by the addition of ABT-737.

## DISCUSSION

We found that DHA and its derivative X-11-induced apoptosis in AML HL-60 and NB4 cells and that this effect was associated with Noxa induction (Fig. 1D, 5B). Overexpression of Mcl-1 (Fig. 5C, 5D) and silencing of Noxa and Bak attenuated X-11-induced apoptosis (Fig. 6A, 6B), supporting the notion that Noxa up-regulation leads to Bak activation through inactivation of Mcl-1. Mcl-1 is a key anti-apoptotic protein that protects mature neutrophils against cell death [38-39]. The activated MEK/ERK and AKT/mTOR signaling pathways have been found to increase Mcl-1 levels in AML cells [40-42] such that Mcl-1 is being considered as a therapeutic target for AML therapy [42]. Although many agents can decrease the levels of Mcl-1 protein, only limited efficacies were observed in AML patients [42]. It is possible that agents inhibiting Mcl-1 activity through other pathways will be more effective and need to be developed. Noxa, a BH3-only protein, specifically binds to Mcl-1 and then leads to Bak activation and apoptosis [9]. Based on the specificity of Noxa binding to Mcl-1, Noxa mimetics are being developed [43]. Several agents approved for cancer therapy such as bortezomib have been found to induce Noxa expression [44].

Although it has been reported that DHA induces Noxa and/or activates Bak in melanoma and lung adenocarcinoma cells, much higher concentrations of DHA were used [23, 45]. Since in AML cells DHA is capable of inducing Noxa expression at 100-fold lower concentrations than those used in solid tumors, it suggests that DHA induces Noxa in AML cells through different mechanisms. We found that several factors mediate the Noxa induction in AML cells. First, we determined that the endoperoxide moiety of DHA and X-11, as well as the intracellular iron, were required for Noxa induction in AML cells (Figs. 3, 4). The connection of iron with the endoperoxide moiety may come from their interaction to form carbon-center radical which mediates apoptosis induction in AML cells [16]. Second, we found that O_2_^−^, but not H_2_O_2_, mediates Noxa induction in AML cells. The role of ROS in DHA-induced apoptosis is controversial [15, 18, 23]. It has been found that Noxa induction in melanoma and Jurkat cells treated with high concentrations of DHA was inhibited by antioxidant NAC [18, 23]. We found that the antioxidant NAC and catalase did not block DHA and X-11-induced apoptosis or Noxa induction (Supp Fig. 2), which is consistent with a previous report showing that DHA-induced apoptosis was not blocked by NAC in HL-60 cells [15]. However, we did find that increase of the O_2_^−^ levels after DHA and X-11 treatment contributes to apoptosis and Noxa induction, and is blocked by an O_2_^−^ inhibitor DPI (Fig. 2). It seems that DHA and X-11 form more active products with iron and, by targeting mitochondrial function, produce more O_2_^−^ [46, 47]. This, in turn, leads to Noxa and apoptosis induction in AML cells. Moreover, we found that FOXO3a is the mediator of increased levels of O_2_^−^ and Noxa protein. FOXO3a is a transcription factor known to induce expression of several pro-apoptotic proteins such as Bim, FasL and TRAIL [48]. There is only one report showing that FOXO3a induces Noxa expression in neuroblastoma cells [31]. We found that FOXO3a is a novel mediator of Noxa-induction by X-11 treatment (Fig. 6C, 6D). Silencing of FOXO3a inhibited X-11-induced up-regulation of Noxa and O_2_^−^ inhibitor DPI blocked X-11 induction of FOXO3a and Noxa up-regulation (Fig. 6E, 6F). Therefore we observe a new cascade of O_2_^−^/FOXO3a/Noxa in AML cells treated with X-11 which plays an essential role in apoptosis induction.

AML cells express Bcl-2 with varied expression of Bcl-xL [32]. HL-60 and NB4 cells which express high levels of Bcl-2 without Bcl-xL, are sensitive to DHA and X-11-induced apoptosis (Figs. 1, 5). Since both cell lines also express high levels of Bim, which binds to Bcl-2, Bcl-xL and Mcl-1, the anti-apoptotic effect of Bcl-2 in those lines is compromised by the expression of Bim. Overexpression of Bcl-2 in HL-60 cells decreases their sensitivity to DHA and X-11-induced apoptosis even when Noxa is induced (Fig. 9). This might be due to Bcl-2 competition for Bim which frees Mcl-1, a process that would require more Noxa to antagonize the anti-apoptotic effects of Mcl-1. Therefore, the ratio between Bcl-2 and Bim could regulate the sensitivity of AML cells to DHA and X-11-induced apoptosis. Although we found that DHA and X-11 weakly induce the Bim isoforms, Bim_L_ and Bim_S_, in HL-60 cells (Fig. 1D), the levels of Bim_L_ and Bim_S_ were not induced in NB4 cells treated with DHA and X-11 (Fig. 5B). These data suggest that the basal, not the induced levels of Bim, are sufficient to antagonize the Bcl-2 and to participate in DHA/X-11-induced apoptosis. Some AML cells, such as U937, express all three anti-apoptotic proteins, Bcl-2, Bcl-xL, and Mcl-1. Bcl-xL also binds to Bak and protects cells from apoptosis in a way similar to that of Mcl-1 [42]. Since Noxa does not bind to Bcl-xL, Bcl-xL needs to be inhibited in AML cells treated with DHA/X-11. ABT-737 is a newly developed Bcl-2 and Bcl-xL inhibitor [49]. The apoptosis induction ability of ABT-737 is attenuated by increased levels of Mcl-1 [34]. ABT-737 acts synergistically with DHA and X-11 to induce apoptosis in U937 cells (Fig. 7, 8). Our data provide a strong rationale for the combined use of DHA with ABT-737 for AML patient treatment with the goal of inhibiting both Mcl-1 and Bcl-xL/Bcl-2.

**Figure 10:**
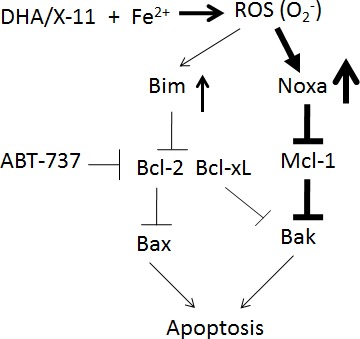
A working model of DHA and X-11 apoptosis induction in AML cells DHA or X-11 interacts with iron to produce ROS (O_2_^−^) through the endoperoxide moiety, which strongly upregulates Noxa and weakly upregulates Bim. The induced Noxa protein binds to Mcl-1 and then leads to Bak activation. The anti-apoptotic effect of Bcl-2 is antagonized by basal and induced levels of Bim. ABT-737 combined with DHA synergistically induces apoptosis in AML cells due to respective inhibition of Bcl-2/Bcl-xL and Mcl-1.

Overall, we found that by inducing Noxa, inactivating Mcl-1 and causing Bak activation, DHA and X-11 are potent apoptosis inducers in AML cells. The weakly induced and/or basally expressed Bim participates in the apoptosis induction by inhibiting Bcl-2. DHA/X-11 in combination with ABT-737 synergistically induce apoptosis in AML cells that express high levels of Bcl-xL and/or Bcl-2. Intracellular iron and the endoperoxide moiety of DHA and X-11 are required for apoptosis and Noxa induction through a ROS-mediated pathway (Fig. 10). Since normal myeloid cells contain much lower levels of iron and are much less sensitive to DHA-induced cell death [46], DHA and X-11 could be developed as selective apoptosis inducers in AML cells alone or in combination with ABT-737.

## MATERIALS AND METHODS

### Reagents

X-11, DODHA (deoxy-dihydroartemisinin), and DOX-11 (10-O-[4-(1-acetyl-5-phenyl-4,5-dihydropyrazol-3-yl) phenyl]-(10S)-deoxy-dihydroartemisinin) were synthesized as described in supp Fig. 3. DHA, acridine orange (AO), ethidium bromide (EB), N-acetylcysteine (NAC) and catalase (CAT), ferrous sulfate (Fe^2+^), ferric ammonium citrate (Fe^3+^), diphenyleneiodonium chloride (DPI) and deferoxamine mesylate (DFO) were purchased from Sigma Chemical Co. (St. Louis, MO). ABT-737 was purchased from Selleckchem (Houston, TX). 5,6-carboxy-2′,7′-dichlorodihydrofluorescein diacetate (DCFH-DA), Rhodamine-123 (Rh123) and MitoSOX™ Red were purchased from Molecular Probes (Eugene, OR). Antibodies to poly-(ADP-ribose)-polymerase (PARP), caspase-3 and caspase-8 were obtained from BD Biosciences (San Diego, CA); to Mcl-1, Bcl-2, Bcl-xL, Bax, and β-actin were from Santa Cruz Biotechology, Inc. (Santa Cruz, CA); to Bim, Bak, CHOP, FOXO3a, Puma, cleaved caspase-9 and -3 were from Cell Signaling Technology, Inc. (Beverly, MA); and to Noxa was from Abcam Inc. (Cambridge, MA).

### Cell lines

Human acute myeloid leukemia HL-60 cells, human acute promyelocytic leukemia NB4 cells, and human acute monocytic leukemia U937 cells, were cultured in RPMI 1640 medium supplemented with 100 units/mL penicillin, 100 μg/mL streptomycin and 10% (v/v) heat-inactivated fetal bovine serum (FBS) [50]. Jurkat cell (sub-clone A3) and its caspase-8 deficient sub-clone, I 9.2 (obtained from ATCC, Rockville, MD), were cultured in RPMI 1640 medium modified to contain 2 mmol/L L-glutamine, 10 mmol/L HEPES, 1.0 mmol/L sodium pyruvate, 4.5 g/L glucose, 1.5 g/L sodium bicarbonate, 100 U/mL penicillin, 100 mg/mL streptomycin, and 10% FBS [51]. HL-60/neo and HL-60/Bcl2 cells are clones of human HL-60 cells transfected with an empty vector and a Bcl-2-expression plasmid (obtained from Dr. Cleary) [52]. HL-60/V3 and HL-60/M15 cells are clones of human HL-60 cells transfected with an empty vector and a Mcl-l-expression plasmid generated by us previously [53].

### Quantitation of apoptotic cells

Apoptotic cells were determined by morphologic observation and fluorescence-activated cell sorting (FACS) analysis after staining with PI or Annexin V [53]. For morphologic apoptosis quantification, cells were stained with AO and EB as described previously [50], and the percentage of apoptotic cells was calculated from 300 cells. For FACS analysis with PI staining, cells were fixed with ice-cold 70% ethanol at a density of 1×10^5^ cells/mL and treated with 1 mg/mL RNase for 30 min at 37 °C. PI was then added to a final concentration of 50 μg/mL and the DNA content was quantitated by flow cytometry (Becton Dickinson, San Jose, CA) with an excitation wavelength of 488 nm and an emission wavelength of 625 nm. Data were analyzed using CELLQuest (Becton Dickinson) software. For FACS analysis with Annexin V staining, Annexin V–FITC Apoptosis Detection Kit and Annexin V–PI Apoptosis Detection Kit (BD Biosciences) were used to quantify apoptosis by FACS analysis.

### Determination of intracellular H_2_O_2_ levels

Intracellular H_2_O_2_ levels were monitored using DCFH-DA by flow cytometry. Briefly, cells (1 × 10^5^ cells/ml) were first mixed with 0.5 μmol/L DCFH-DA for 1 h and then incubated with or without DHA and X-11 for various time periods at 37°C. After washing with PBS, cells were analyzed by flow cytometry with excitation and emission wavelengths of 495 nm and 525 nm, respectively [50].

### Measurement of intracellular superoxide anion (O_2_^−^) content

Levels of intracellular O_2_^−^ content were measured by mitochondrial superoxide indicator MitoSOX™ Red, which rapidly enters live cells and is oxidized by superoxide, but not by other ROS- or reactive nitrogen species (RNS)–generating systems, and exhibits red fluorescence. Briefly, cells (1 × 10^5^ cells/ml) treated or untreated with DHA/X-11 for various time periods, were incubated with 5 μmol/L MitoSOX™ Red for 15 min at 37°C, protected from light. After washing with phosphate buffer saline (PBS), cells were analyzed by flow cytometry with excitation and emission wavelength of 510 and 580 nm, respectively.

### Western blot analysis

Protein extracts (40 μg) prepared with RIPA lysis buffer [50 mmol/L Tris-HCl, 150 mmol/L NaCl, 0.1% SDS, 1% NP40, 0.5% sodium deoxycholate, 1 mmol/L phenylmethylsulfonyl fluoride (PMSF), 100 μmol/L leupeptin, and 2 μg/mL aprotinin (pH, 8.0)] were separated on 10% or 15% sodium dodecyl sulfate (SDS)-polyacrylamide gels and transferred to nitrocellulose membranes. After blocking with 5% nonfat milk, the membranes were incubated with specific antibodies overnight at 4°C. Immunocomplexes were visualized using enhanced chemiluminescence Western blotting detection reagents (Amersham Biosciences Inc., Piscataway, NJ) [50].

### RNA interference

*NOXA*, *BAK, FOXO3A* siRNA and a control siRNA were purchased from Santa Cruz Biotechnology, Inc. siRNAs were transfected into NB4 cells with a nucleofector (Amaxa, Gaithersburg, MD) following the manufacturer's instructions. Briefly, 2×10^6^ cells were electroporated in 100 μL nucleofector solution (Amaxa Reagent V) with siRNA (200 pmol), using preselected Amaxa Program T-003. Cells were plated in 6-wells plates with 2 ml supplemented RPMI-1640 medium for 18 h at 37°C, and then were treated with X-11 for further 18 h. The cells were harvested and used for FACS and Western blotting analysis.

### Reverse transcription-PCR

RNA isolation was performed using the RNA isolation kit (Gentra). cDNA was prepared using an oligo(dT) primer and SuperScript II Reverse Tanscriptase (Invitrogen) following standard protocols. Primers used in these experiments were as follows: *NOXA*, 5′-AGAAGGCGCGCAAGAACGCT-3′ and 5′-TTTCTCCCCAGCCGCCCAGT-3′; and *β-actin*, 5′-ATCTGGCACCACACCTTCTACAATGAGCTGCG-3′ and 5′-CGTCATACTCCTGCTTGCTGATCCACATC TGC-3′. After reverse transcription, the cDNA product was amplified by PCR with two units of *Taq* DNA polymerase (Invitrogen) using standard protocols at annealing temperature of 55°C.

## SUPPLEMENTARY MATERIAL, FIGURES


