# Supplementary material for: Dihydroartemisinin and its derivative induce apoptosis in acute myeloid leukemia through Noxa-mediated pathway requiring iron and endoperoxide moiety
Source: Oncotarget. 2015 Jan 21;6(8):5582–96. doi: 10.18632/oncotarget.3336 (PMC4467388; doi:10.18632/oncotarget.3336)
Supplement: Supplementary file 1 [file oncotarget-06-5582-s001.pdf]

Dihydroartemisinin and its derivative induce apoptosis in acute myeloid leukemia through Noxa-mediated pathway requiring iron and endoperoxide moiety

Supplementary Material

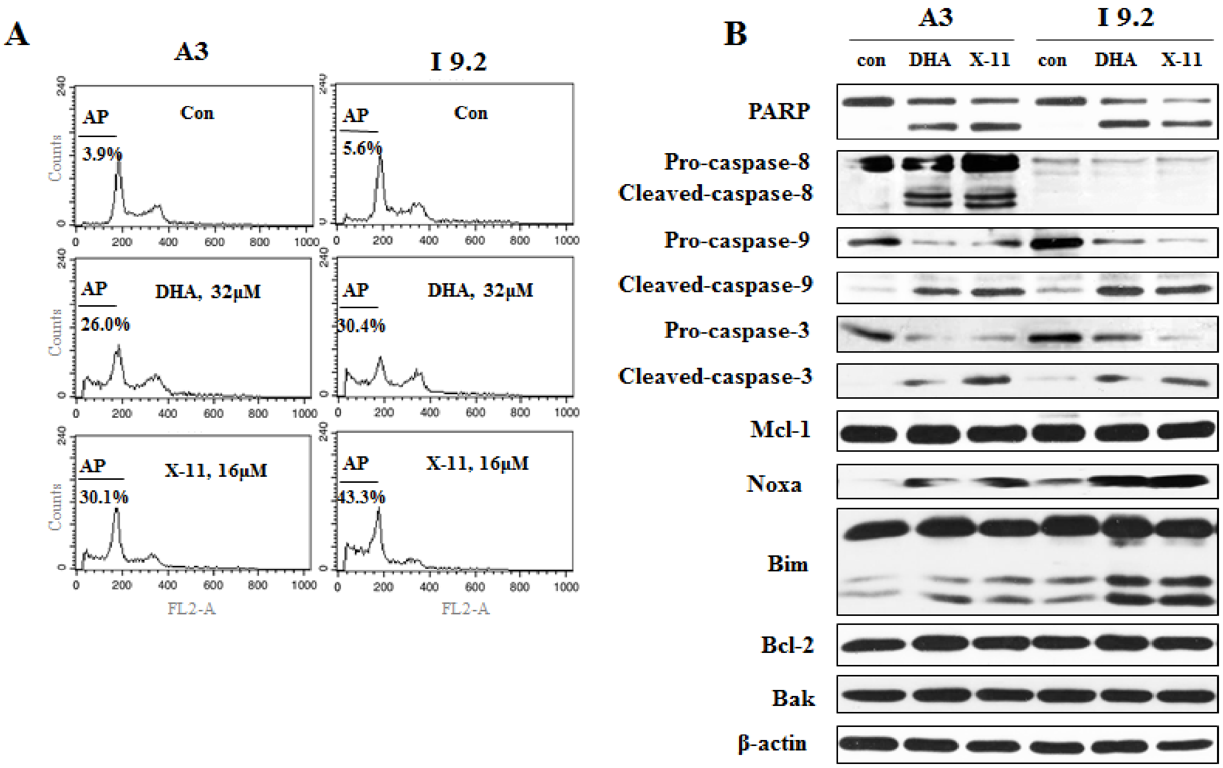

Supplementary Figure 1: Jurkat subclones with or without caspase-8 expression are equally sensitive to DHA and X-11-induced apoptosis. A3 cells expressing caspase-8 and I 9.2 cells lacking caspase-8 expression were treated with DHA or X-11 at the indicated concentrations for 24 h. The percentage of apoptotic cells was determined by FACS after staining with PI (A) and the apoptosis related proteins were measured by Western blot analysis (B).

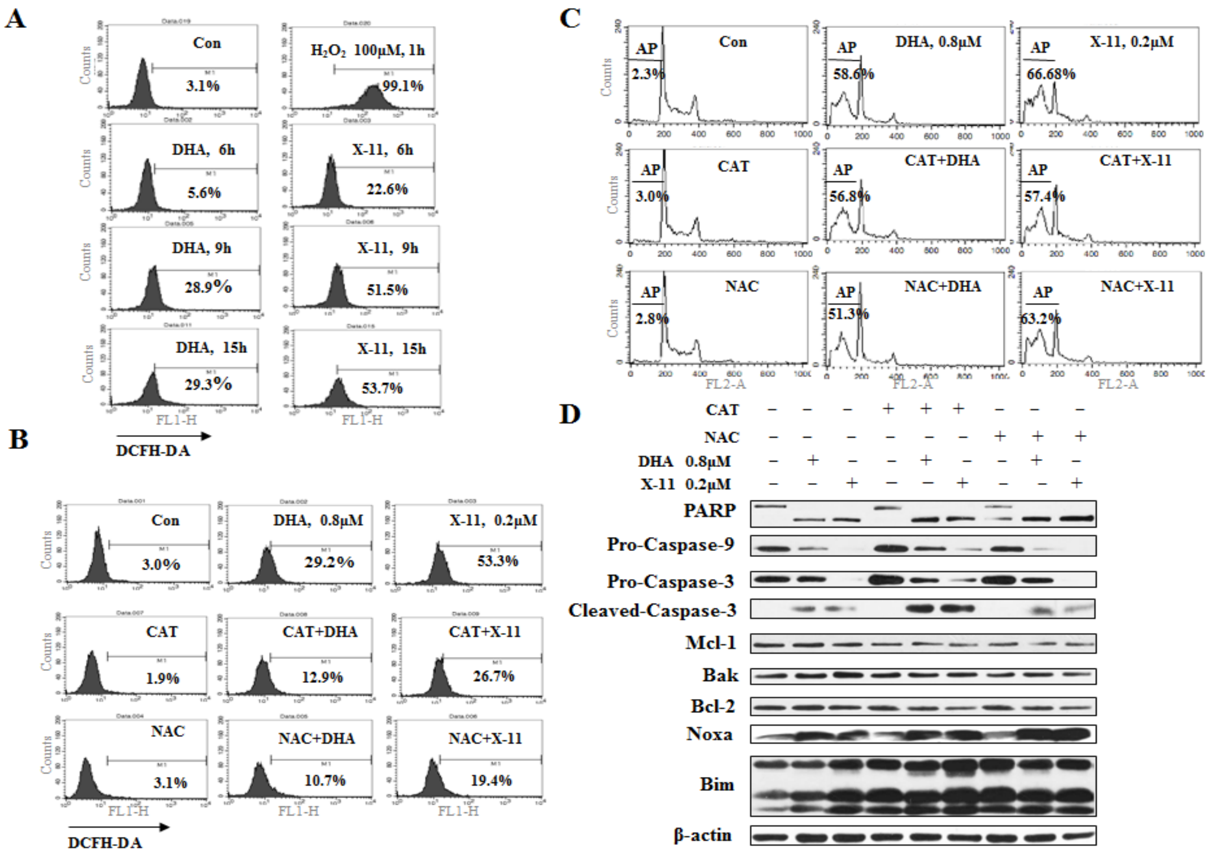

Supplementary Figure 2: Antioxidants NAC and CAT did not block DHA/X-11-induced apoptosis in HL-60 cells. A, H_2O_2 content measured with DCFH-DA. HL-60 cells were labeled with 5 μM DCFH-DA fluorescent probe for 1 h and then treated with or without 0.8 μM DHA or 0.2 μM X-11 for different times. Oxidized DCF levels were analyzed using FACS. H_2O_2 at 100 μM for 1 h treatment was used as a positive control. Shift of the peak to the right indicates increased levels of H_2O_2 content. B, inhibition of DHA/X-11 induced H_2O_2 production by NAC and CAT. HL-60 cells were pretreated with or without 10 mM NAC or 500 units/ml CAT, followed by treatment with 0.8 μM DHA or 0.2 μM X-11 for 15 h, and the H_2O_2 contents were measured. C & D, the effects NAC and CAT on DHA and X-11-induced apoptosis and changes of apoptosis related proteins. HL-60 cells were pretreated with and without 500 units/ml CAT or 10 mM NAC for 4 h, followed by treatment with or without 0.8 μM DHA or 0.2 μM X-11 for 24 h. Percentages of apoptotic cells were determined using FACS after PI staining (C) and the protein levels were measured by Western blot analysis (D).

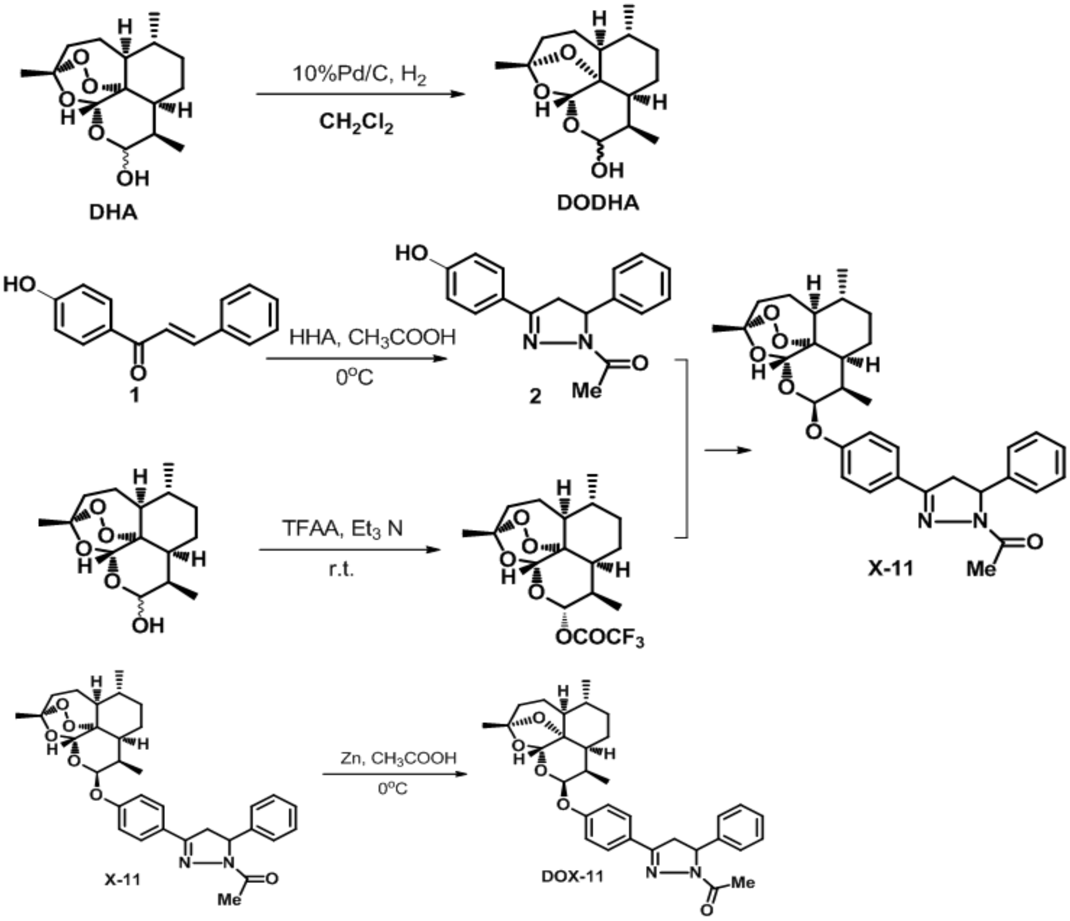

HHA: hydrazine hydrate; TFAA: trifluoroacetic anhydride.

Supplementary Figure 3: The synthetic routes of X-11, DODHA and DOX-11. To obtain the X-11, compound 2 was synthesized from chalcone 1, thus reacted with DHA-trifluoroacetate. To give DODHA, DHA was subjected to a reduction by H_2 and Pd/C while X-11 synthesized from chalcone was transformed to DOX-11 using Zn/acetate acid as reductant. The chemical structures of DODHA, X-11 and DOX-11 were determined by mass spectrometry (MS) and ^{1}H nuclear magnetic resonance (NMR).

Data for DODHA are as follows: MS: $(M+Na)^+$ 291.1, $(2M+Na)^+$ 259.3;^{1}H-NMR (DMSO, δ (ppm)): 0.82(t, 6H, H-15, H-16), 1.43(s, 3H, H-14), 2.35(m, 1H, H-9), 5.14(m, 1H, H-10), 5.28(s, 1H, H-12), 6.27(d, 1H, OH-10).

Data for X-11 are as follows: MS: $(M+H)^+$ 547.2;^{1}H-NMR (DMSO, δ (ppm)): 0.88(d, 3H, J=6.3, H-15), 0.97(d, 3H, J=7.2, H-16), 1.28(s, 3H, H-14), 2.17(m, 1H, H-4), 2.28(s, 3H, $COC\underline{H}_3$), 2.59(m, 1H, H-9), 3.10(d, 1H, $C\underline{H}_2CH$), 3.81(m, 1H, $C\underline{H}_2CH$), 5.37(s, 1H, $CH_2C\underline{H}$), 5.50(s, 1H, H-12), 5.64(d, 1H, J=3.3, H-10), 6.63(d, 1H, J=8.4, Ar-H), 7.13(d, 2H, J=8.4, Ar-H), 7.16(d, 2H, J=7.8, Ar-H), 7.23(m, 1H, Ar-H), 7.31(d, 2H, J=7.8, Ar-H), 7.73(d, 2H, J=8.4, Ar-H).

Data for DOX-11 are as follows: MS: $(M+H)^+$ 531.1;^{1}H-NMR (DMSO, δ (ppm)): 0.84(d, 3H, *J*=6.3, H-15), 0.99(d, 3H, *J*=7.2, H-16), 1.46(s, 3H, H-14), 2.29(s, 3H, $COC\underline{H}_3$), 3.06(d, 1H, $C\underline{H}_2CH$), 3.81(m, 1H, $C\underline{H}_2CH$), 5.39(s, 1H, $CH_2C\underline{H}$), 5.52(s, 1H, H-12), 5.62(d, 1H, *J*=3.3, H-10), 7.11(d, 1H, *J*=8.4, Ar-H), 7.17(d, 2H, *J*=8.4, Ar-H), 7.25(d, 2H, *J*=7.8, Ar-H), 7.30(m, 1H, Ar-H), 7.33(d, 2H, *J*=7.8, Ar-H), 7.72(d, 2H, *J*=8.4, Ar-H).

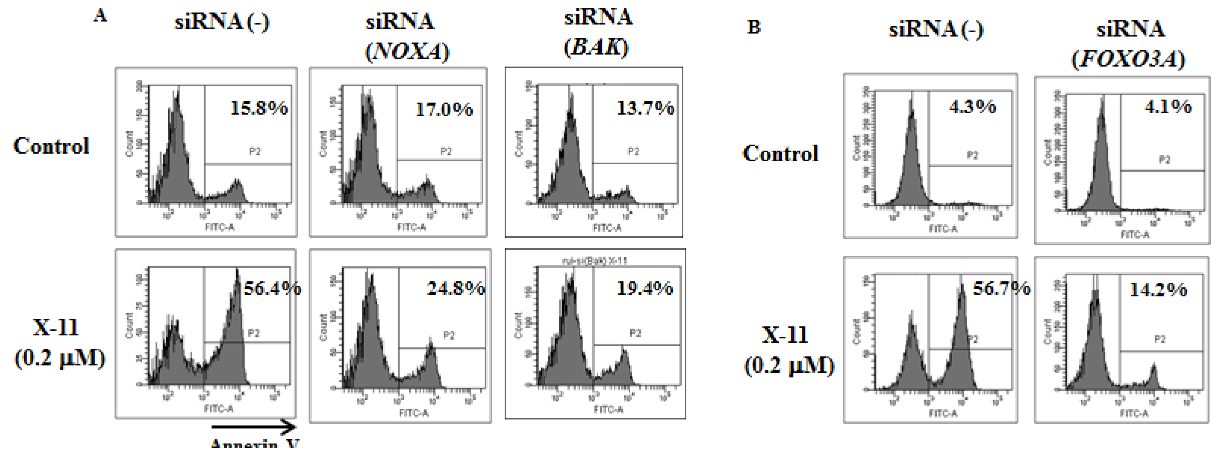

Supplementary Figure 4: Silencing of Noxa, Bak or FOXO3a attenuated X-11-induced apoptosis in NB4 cells. NB4 cells were transfected with *NOXA, BAK or FOXO3A* siRNA or a negative control siRNA, and after 18 h, treated with 0.2 µM X-11 for 18 h. The apoptotic cells were determined by FACS after staining with annexin V- fluorescein isothiocyanate (FITC).
